# An Accurate Upper Bound Solution for Plane Strain Extrusion through a Wedge-Shaped Die

**DOI:** 10.1155/2014/189070

**Published:** 2014-06-29

**Authors:** Sergei Alexandrov, Yusof Mustafa, Yeong-Maw Hwang, Elena Lyamina

**Affiliations:** ^1^A. Yu. Ishlinskii Institute for Problems in Mechanics, Russian Academy of Sciences, 101-1 Prospect Vernadskogo, Moscow 119526, Russia; ^2^Faculty of Mechanical Engineering, Universiti Teknologi Malaysia, 81310 Skudai, Johor Darul Ta'zim, Malaysia; ^3^Department of Mechanical and Electro-Mechanical Engineering, National Sun Yat-sen University, Kaohsiung 804, Taiwan

## Abstract

An upper bound method for the process of plane strain extrusion through a wedge-shaped die is derived. A technique for constructing a kinematically admissible velocity field satisfying the exact asymptotic singular behavior of real velocity fields in the vicinity of maximum friction surfaces (the friction stress at sliding is equal to the shear yield stress on such surfaces) is described. Two specific upper bound solutions are found using the method derived. The solutions are compared to an accurate slip-line solution and it is shown that the accuracy of the new method is very high.

## 1. Introduction

The approximate methods for analysis of plane strain and axisymmetric extrusion/drawing can be conveniently divided into three groups: (i) the slab method, (ii) semianalytical solutions for flow through infinite channels, and (iii) the upper bound method. The slab method has been used, for example, in [1]. In general, this method is oversimplified. In particular, the through-thickness distribution of stress and strain rate is completely ignored. Semianalytical solutions for flow of plastic materials through infinite converging channels based on various simplifications have been proposed in [2–8] for several rigid plastic models. A disadvantage of the application of these solutions to the process of extrusion is that they do not account for the power dissipation at velocity discontinuity surfaces. Therefore, the upper bound method is probably the best approximate method for rapid analysis and design of metal forming processes. Upper bound solutions for axisymmetric and plane strain extrusion have been proposed in [9, 10], respectively. The accuracy of predictions based on the upper bound method strongly depends on the kinematically admissible velocity field chosen. General kinematically admissible velocity fields suitable for analysis of metal forming processes have been proposed in [11–15]. A disadvantage of these kinematically admissible velocity fields is that they do not account for the singular asymptotic behavior of real velocity fields in the vicinity of maximum friction surfaces (the friction stress at sliding is equal to the shear yield stress on such surfaces) found in [16]. Even though this asymptotic behavior is not a formal requirement of the upper bound theorem, it is advantageous to take it into account in kinematically admissible velocity fields. This has been demonstrated in applications of the upper bound method to analysis of welded structures [17] and several metal forming processes [18–20]. One of the present authors proposed a set of stream functions to investigate the plastic deformation behavior of the rods during axisymmetric extrusion of composite rods through a conical die [21]. In the present paper, a general kinematically admissible velocity field for the process of plane strain extrusion through a wedge-shaped die is built up. This velocity field satisfies the asymptotic behavior found in [16]. Two upper bound solutions are found using the method proposed. The velocity field from an exact semianalytical solution for plastic flow through an infinite wedge-shaped channel derived in [22] is adopted as one of the kinematically admissible velocity fields. The other solution is based on a very simple kinematically admissible velocity field. The solutions are compared to a slip-line solution found by means of the method of Riemann in [23]. A very close agreement between all three solutions confirms the high accuracy of the method proposed.

## 2. General Considerations

**Figure 1:**
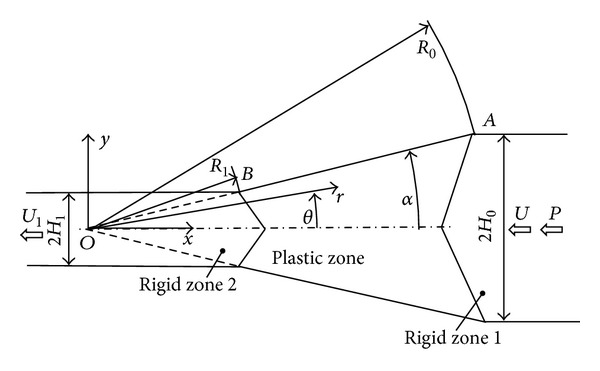
Geometry of the process.

Consider a wedge-shaped die (total angle 2*α*) through which a sheet of plastic material is being pushed by force *P* (Figure 1). The initial thickness of the sheet is 2*H*_0_ and its final thickness is 2*H*_1_. There are two rigid zones and one plastic zone. The shape of the rigid/plastic boundaries is unknown and should be found from the solution. The speed of rigid zone 1 is *U* and the speed of rigid zone 2 is *V*. Let polar coordinates (*r*, *θ*) be taken relative to the axis of symmetry and the virtual apex *O* of the die. In addition, introduce Cartesian coordinates (*x*, *y*) whose origin is situated at *O* and whose* x*-axis coincides with the axis *θ* = 0. In the polar coordinates, the surfaces of the die are determined by the equation *θ* = ±*α*. Since *θ* = 0 is an axis of symmetry for the flow, it is sufficient to find the solution in the region *θ* ≥ 0. It is assumed that the sheet is rigid perfectly/plastic and obeys Mises yield criterion. The tensile yield stress is denoted by *σ*_0_.

The nonzero strain rate components in the polar coordinate system are *ξ*_*rr*_, *ξ*_*θθ*_, and *ξ*_*rθ*_. The equivalent strain rate is defined by

(1) $$\begin{array}{c} \xi_{\text{eq}}=\sqrt{\frac{2}{3}}{\left(\xi_{rr}^{2}+\xi_{\theta \theta }^{2}+2\xi_{r\theta }^{2}\right)}^{1/2}. \end{array}$$

The surfaces of the die are rough and it is supposed that the frictional stress *τ*_*f*_ is constant. Therefore,

(2) $$\begin{array}{c} \tau_{f}=\frac{m\sigma_{0}}{\sqrt{3}},\;m\leq 1, \end{array}$$

at *θ* = *α*. The maximum friction law is obtained if *m* = 1. A distinguished feature of this law is that the material cannot support a shear stress larger than $\sigma_{0}/\sqrt{3}$. A distinguished mathematical feature of solutions in the vicinity of maximum friction surfaces is that the equivalent strain rate approaches infinity near such surfaces. In particular [16],

(3) $$\begin{array}{c} \xi_{\text{eq}}=Ds^{-1/2}+o\left(s^{-1/2}\right) \end{array}$$

as *s* → 0. Here *s* is the normal distance to the maximum friction surface and *D* is the strain rate intensity factor. Equation (3) describes the behavior of real velocity fields. It is not necessary but advantageous to account for this behavior of the equivalent strain rate in kinematically admissible velocity fields. Moreover, it has been demonstrated in [18] that using (3) leads to more accurate upper bound solutions, as compared to other kinematically admissible velocity fields of the same level of complexity, even if *m* < 1. The circumferential velocity must vanish at *θ* = *α*.

## 3. Kinematically Admissible Velocity Field

Assume that the circumferential velocity vanishes everywhere. Then, the velocity boundary condition at *θ* = *α* is automatically satisfied. The equation of incompressibility in the polar coordinates reduces to ∂*u*/∂*r* + *u*/*r* = 0. Here *u* is the radial velocity. The general solution of the incompressibility equation is

(4) $$\begin{array}{c} u=-UR_{0}\frac{f\left(\theta \right)}{r}. \end{array}$$

Here *f*(*θ*) is an arbitrary function of *θ* and *R*_0_ is the radial coordinate of point *A* (Figure 1). The nonzero strain rate components are found from (4) as

(5) $$\begin{array}{c} \xi_{rr}=UR_{0}\frac{f\left(\theta \right)}{r^{2}},\;\;\xi_{\theta \theta }=-UR_{0}\frac{f\left(\theta \right)}{r^{2}},\\ \xi_{r\theta }=-\frac{UR_{0}}{2r^{2}}\frac{df}{d\theta }. \end{array}$$

Substituting (5) into (1) yields

(6) $$\begin{array}{c} \xi_{\text{eq}}=\frac{UR_{0}}{\sqrt{3}r^{2}}\sqrt{4f^{2}+{\left(\frac{df}{d\theta }\right)}^{2}}. \end{array}$$

**Figure 2:**
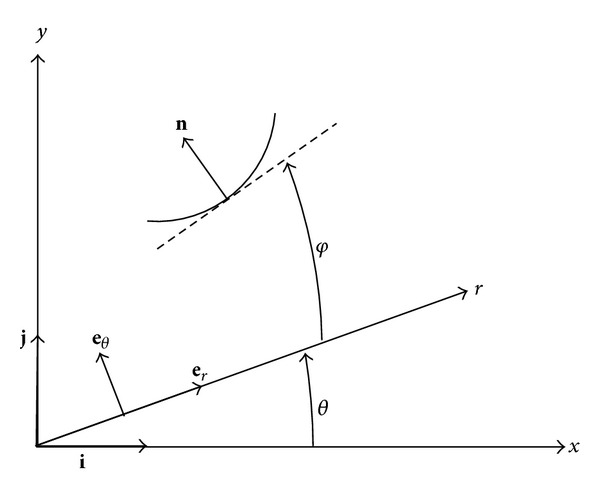
Geometry of a generic velocity discontinuity line.

In order for the velocity field (4) to be compatible with the motion of rigid zones 1 and 2 (Figure 1), it is necessary to introduce velocity discontinuity lines through points *A* and *B*. The normal velocity must be continuous across these velocity discontinuity lines. Let **e**_*r*_ and **e**_*θ*_ be the unit base vectors of the polar coordinates and let **i** and **j** be the unit base vectors of the Cartesian coordinates. Consider an arbitrary velocity discontinuity line (Figure 2). Let **n** be the unit normal vector to this line at its generic point. Then,

(7) $$\begin{array}{c} n=-\sin\varphi e_{r}+\cos\varphi e_{\theta }. \end{array}$$

Here *φ* is the orientation of the tangent to the velocity discontinuity line relative to the *r*-axis at the same point. The velocity of each of the rigid zones can be represented in the following form:

(8) $$\begin{array}{c} u_{r}=-vi. \end{array}$$

where *v* = *U* for rigid zone 1 and *v* = *V* for rigid zone 2. With no loss of generality, it is possible to assume that the value of *U* is prescribed. Then, it follows from the equation of incompressibility that

(9) $$\begin{array}{c} V=\frac{U}{h},\;\;h=\frac{H_{1}}{H_{0}}. \end{array}$$

The base vector **i** is represented as (Figure 2)

(10) $$\begin{array}{c} i=e_{r}\cos\theta -e_{\theta }\sin\theta . \end{array}$$

Substituting (10) into (8) yields

(11) $$\begin{array}{c} u_{r}=-v\left(e_{r}\cos\theta -e_{\theta }\sin\theta \right). \end{array}$$

The velocity vector in the plastic zone is

(12) $$\begin{array}{c} u_{p}=ue_{r}. \end{array}$$

The condition of continuity of the normal velocity across the velocity discontinuity line can be written as

(13) $$\begin{array}{c} u_{p}\cdot n=u_{r}\cdot n. \end{array}$$

Substituting (4), (7), (11), and (12) into (13) gives

(14) $$\begin{array}{c} tf\left(\theta \right)\sin\varphi =\rho \left(\sin\varphi \cos\theta +\sin\theta \cos\varphi \right), \end{array}$$

where *t* = *U*/*v* *и* *ρ* = *r*/*R*_0_. It follows from the geometry of Figure 2 that

(15) $$\begin{array}{c} \tan\varphi =\frac{rd\varphi }{dr}. \end{array}$$

Substituting (15) into (14) leads to the following differential equation for the velocity discontinuity line:

(16) $$\begin{array}{c} \frac{d\rho }{d\theta }\sin\theta +\rho \cos\theta =tf\left(\theta \right). \end{array}$$

The general solution of this equation is

(17) $$\begin{array}{c} \rho =\rho_{d}\left(\theta \right)=\frac{1}{\sin\theta }\left(C+t\overset{\theta }{\underset{0}{\int }}f\left(z\right)dz\right). \end{array}$$

Here *C* is a constant of integration and *z* is a dummy variable of integration. It is natural to require that the velocity discontinuity line intersects the axis *θ* = 0. Then, the function *ρ*_*d*_(*θ*) must tend to a finite limit as *θ* → 0. A necessary condition for that is *C* = 0. Then, it follows from (17) that

(18) $$\begin{array}{c} \rho =\rho_{d}\left(\theta \right)=\frac{t}{\sin\theta }\overset{\theta }{\underset{0}{\int }}f\left(z\right)dz. \end{array}$$

Since *t* = 1 for the velocity discontinuity line between the plastic zone and rigid zone 1 and *t* = *h* for the velocity discontinuity line between the plastic zone and rigid zone 2, the equations for these lines are obtained from (18) as

(19) $$\begin{array}{c} \rho =\rho_{d0}\left(\theta \right)=\frac{1}{\sin\theta }\overset{\theta }{\underset{0}{\int }}f\left(z\right)dz,\\ \rho =\rho_{d1}\left(\theta \right)=\frac{h}{\sin\theta }\overset{\theta }{\underset{0}{\int }}f\left(z\right)dz, \end{array}$$

respectively. The line *ρ* = *ρ*_*d*0_(*θ*) passes through point *A* and the line *ρ* = *ρ*_*d*1_(*θ*) passes through point *B* (Figure 1). Therefore, *ρ*_*d*0_(*α*) = 1 and *ρ*_*d*1_(*α*) = *h*. Substituting these conditions into (19) gives the following restriction on the function *f*(*θ*):

(20) $$\begin{array}{c} \sin\alpha =\overset{\alpha }{\underset{0}{\int }}f\left(\theta \right)d\theta . \end{array}$$

Taking into account (11) and (12), the amount of velocity jump across the velocity discontinuity lines is determined as

(21) $$\begin{array}{c} \left[u\right]={\left[{\left(u+v\cos\theta \right)}^{2}+v^{2}\text{si}{\text{n}}^{2}\theta \right]}^{1/2}. \end{array}$$

Substituting (4) into (21) and taking into account that *ρ* = *ρ*_*d*_(*θ*) on the velocity discontinuity lines yield

(22) $$\begin{array}{c} \left[u\right]=\frac{U}{t\rho }{\left[t^{2}f^{2}\left(\theta \right)-2tf\left(\theta \right)\rho_{d}\left(\theta \right)\cos\theta +\rho_{d}^{2}\left(\theta \right)\right]}^{1/2}. \end{array}$$

An infinitesimal length element along the velocity discontinuity lines is represented as

(23) $$\begin{array}{c} dL=R_{0}{\left[{\left(\frac{d\rho_{d}}{d\theta }\right)}^{2}+\rho_{d}^{2}\left(\theta \right)\right]}^{1/2}d\theta . \end{array}$$

Eliminating here the derivative *dρ*_*d*_/*dθ* by means of (16) results in

(24) $$\begin{array}{c} dL=\frac{R_{0}}{\sin\theta }{\left[t^{2}f^{2}\left(\theta \right)-2tf\left(\theta \right)\rho_{d}\left(\theta \right)\cos\theta +\rho_{d}^{2}\left(\theta \right)\right]}^{1/2}d\theta . \end{array}$$

Here, *ρ*_*d*_(*θ*) should be replaced with *ρ*_*d*0_(*θ*) for the velocity discontinuity line between the plastic zone and rigid zone 1 and with *ρ*_*d*1_(*θ*) for the velocity discontinuity line between the plastic zone and rigid zone 2.

## 4. Calculation of the Power Dissipation

The power dissipation per unit length within the plastic zone is determined as

(25) $$\begin{array}{c} W_{V}=\sigma_{0}\overset{\alpha }{\underset{0}{\int }}\overset{R_{0}\rho_{d0}(\theta )}{\underset{R_{0}\rho_{d1}\left(\theta \right)}{\int }}\xi_{eq}r\;dr\;d\theta . \end{array}$$

Substituting (6) into (25) yields

(26) $$\begin{array}{c} W_{V}=\frac{\sigma_{0}UR_{0}}{\sqrt{3}}\overset{\alpha }{\underset{0}{\int }}\overset{\rho_{d0}(\theta )}{\underset{\rho_{d1}\left(\theta \right)}{\int }}\sqrt{4f^{2}+{\left(\frac{df}{d\theta }\right)}^{2}}\rho^{-1}d\rho \;d\theta . \end{array}$$

Since the function *f*(*θ*) is independent of *ρ*, integrating with respect to *ρ* and using (19) result in

(27) $$\begin{array}{c} W_{V}=-\frac{\sigma_{0}UR_{0}\ln h}{\sqrt{3}}\overset{\alpha }{\underset{0}{\int }}\sqrt{4f^{2}+{\left(\frac{df}{d\theta }\right)}^{2}}d\theta . \end{array}$$

The power dissipation per unit length at each of the velocity discontinuity lines is found by means of (19), (22), and (24) as

(28) Wd=σ03∫0α[u]dL=σ0UR03∫0α[f2(θ)F(θ)−2cot θf(θ)+F(θ)sin2θ]dθ,

where *F*(*θ*) = ∫_0_^*θ*^ *f*(*z*)*dz*. It is evident from this equation that the value of *W*_*d*_ is the same for each of the velocity discontinuity lines. The radial velocity at the friction surface is found from (4) as *u*|_*θ*=*α*_ = −*UR*_0_ *f*(*α*)*r* ^−1^. Then, using (2), the power dissipation per unit length at the friction surface is determined in the form

(29) $$\begin{array}{c} W_{f}=-\frac{m\sigma_{0}}{\sqrt{3}}\overset{R_{0}}{\underset{R_{1}}{\int }}{u|}_{\theta =\alpha }dr=-\frac{m\sigma_{0}UR_{0}f\left(\alpha \right)}{\sqrt{3}}\ln h. \end{array}$$

Here the relations *H*_0_ = *R*_0_sin*α* and *H*_1_ = *R*_1_sin*α* that follow from the geometry of Figure 1 have been used. The total power dissipation per unit length for the process *W*_*i*_ can be calculated by summing the various components

(30) $$\begin{array}{c} W_{i}=W_{V}+2W_{d}+W_{f}. \end{array}$$

The factor of two at *W*_*d*_ is to take into account that there are two velocity discontinuity lines. According to the upper bound theorem [22]

(31) $$\begin{array}{c} \frac{P_{u}U}{2B}=W_{i}, \end{array}$$

where *B* is the width of the sheet and *P*_*u*_ is an upper bound on *P*. The values for the various components involved in (30) and, then, in (31) are given by (27), (28), and (29). Therefore,

(32) pu=3Pu2σ0BH0=−ln⁡hsinα[mf(α)+∫0α4f2+(dfdθ)2dθ] +2sinα∫0α[f2(θ)F(θ)−2cot θf(θ)+F(θ)sin2θ]dθ,

where *p*_*u*_ is the dimensionless upper bound on *P*. It is convenient to rewrite (32) in the following form:

(33) $$\begin{array}{c} p_{u}=-G_{0}\left(\alpha \right)\ln h+G_{1}\left(\alpha \right), \end{array}$$

where

(34) $$\begin{array}{c} G_{0}\left(\alpha \right)=\frac{1}{\sin\alpha }\left[mf\left(\alpha \right)+\overset{\alpha }{\underset{0}{\int }}\sqrt{4f^{2}+{\left(\frac{df}{d\theta }\right)}^{2}}d\theta \right],\\ G_{1}\left(\alpha \right)=\frac{2}{\sin\alpha }\overset{\alpha }{\underset{0}{\int }}\left[\frac{f^{2}\left(\theta \right)}{F\left(\theta \right)}-2\text{cot}\;\theta f\left(\theta \right)+\frac{F\left(\theta \right)}{\text{si}{\text{n}}^{2}\theta }\right]d\theta . \end{array}$$

## 5. Examples of the Use of the Method

An exact semianalytical solution for flow of plastic material through an infinite wedge-shaped converging channel has been proposed in [22]. This velocity field can be used as a kinematically admissible velocity field to find *p*_*u*_ by means of (33) and (34). An advantage of this velocity field is that there exists an associated stress field satisfying the yield criterion and the equilibrium equations. Since the aforementioned solution is exact, it automatically satisfies (3). The radial velocity is given by

(35) $$\begin{array}{c} u=-\frac{UR_{0}}{r}\frac{\beta }{\left(c-\cos2\psi \right)}. \end{array}$$

Here *β* and *c* are constant and *ψ* is a function of *θ*. The equation for the function *ψ*(*θ*) is

(36) $$\begin{array}{c} \frac{d\psi }{d\theta }=\frac{c-\cos2\psi }{\cos2\psi }. \end{array}$$

It follows from the stress solution (see [22]) that *τ*_*rθ*_ = *k*sin2*ψ*, where *τ*_*rθ*_ is the shear stress in the polar coordinates. Then, it follows from (2) that

(37) $$\begin{array}{c} \psi_{\alpha }=\frac{1}{2}\arcsin m, \end{array}$$

where *ψ*_*α*_ is the value of *ψ* at *θ* = *α*. Moreover, it follows from the same relation that *ψ* = 0 at *θ* = 0. The solution of (36) satisfying this condition can be written as

(38) $$\begin{array}{c} \theta =\overset{\psi }{\underset{0}{\int }}\frac{\cos2\chi }{\left(c-\cos2\chi \right)}d\chi . \end{array}$$

Here *χ* is a dummy variable of integration. Using (37) and (38), the equation for *c* is obtained in the form

(39) $$\begin{array}{c} \alpha =\overset{\psi_{\alpha }}{\underset{0}{\int }}\frac{\cos2\chi }{\left(c-\cos2\chi \right)}d\chi . \end{array}$$

This equation should be solved numerically for given values of *α* and *m*. The material flux is prescribed, *Q* = *UBH*_0_. Then, it follows from (35) that

(40) $$\begin{array}{c} UBR_{0}\beta \overset{\alpha }{\underset{0}{\int }}\frac{d\theta }{\left(c-\cos2\psi \right)}=UBH_{0}. \end{array}$$

Using (36) to replace integration with respect to *θ* with integration with respect to *ψ* and the geometric relation *H*_0_ = *R*_0_sin*α* (Figure 1), (40) is transformed to

(41) $$\begin{array}{c} \beta =\sin\alpha {\left[\overset{\psi_{\alpha }}{\underset{0}{\int }}\frac{\cos2\psi }{{\left(c-\cos2\psi \right)}^{2}}d\psi \right]}^{-1}. \end{array}$$

Once the value of *c* has been found from (39), the value of *β* is determined from (41) by integration. Comparing (4) and (35) shows that

(42) $$\begin{array}{c} f\left(\theta \right)=\frac{\beta }{\left(c-\cos2\psi \right)}. \end{array}$$

Then, using (36)

(43) $$\begin{array}{c} \frac{df\left(\theta \right)}{d\theta }=-\frac{2\beta \tan2\psi }{\left(c-\cos2\psi \right)}, \end{array}$$

(44) F(θ) =∫0θf(z)dz =β∫0ψcos⁡2χ(c−cos⁡2χ)2dχ =β(c2−1)[arctan((c+1)/(c−1)tanψ)c2−1      +csin2ψ2(c−cos⁡2ψ)].

It follows from (41) and (44) that

(45) ∫0αf(z)dz =F(α) =sinα[∫0ψαcos⁡2ψ(c−cos⁡2ψ)2dψ]−1∫0ψαcos⁡2χ(c−cos⁡2χ)2dχ =sinα.

**Figure 3:**
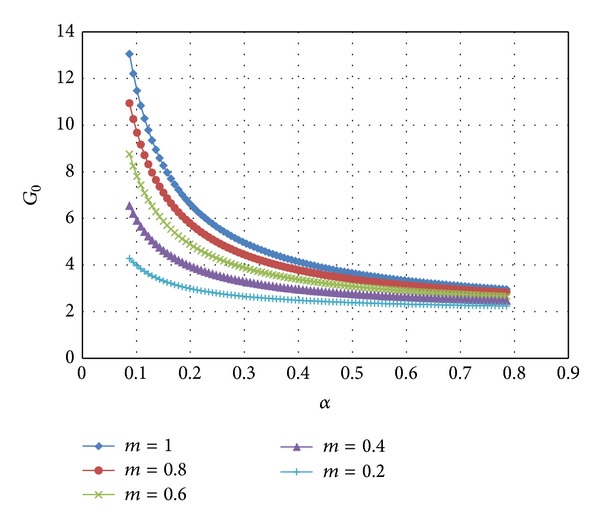
Variation of *G*_0_ with *α* at several values of *m*.

**Figure 4:**
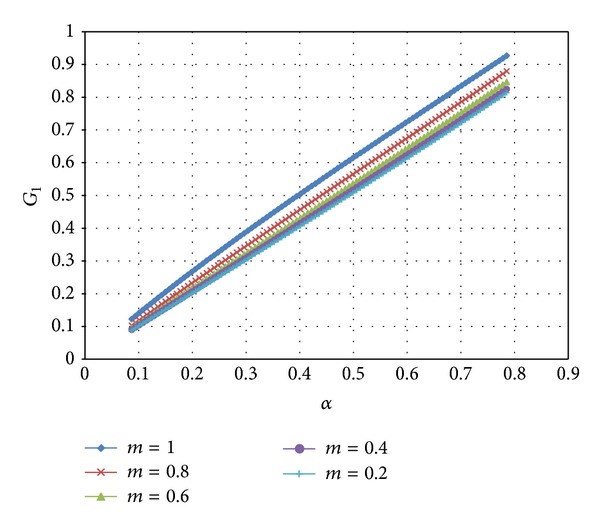
Variation of *G*_1_ with *α* at several values of *m*.

**Figure 5:**
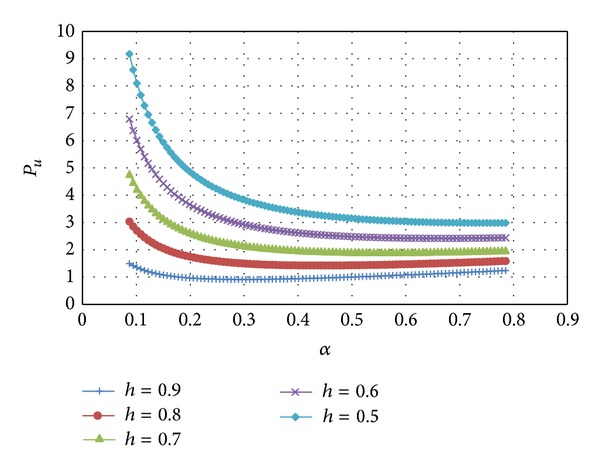
Variation of the dimensionless upper bound extrusion force with *α* at several values of *h* and *m* = 1.

Therefore, (20) is satisfied. Substituting (42)–(44) into (34) the coefficients *G*_0_(*α*) and *G*_1_(*α*) are determined by numerical integration. Then, *p*_*u*_ is readily found from (33). The variation of *G*_0_(*α*) and *G*_1_(*α*) with *α* at several *m*-values is illustrated in Figures 3 and 4, respectively. It is seen from Figure 4 that *G*_1_(*α*) is practically a linear function of *α*. The variation of *p*_*u*_ with *α* at several *h*-values and *m* = 1 is depicted in Figure 5. In order to verify the accuracy of the solution found this value of *p*_*u*_ has been compared to an accurate slip-line solution derived in [23] by the method of Riemann. In particular, the variation of the extrusion pressure with 1 − *h* at several values of *α* (in the nomenclature of the present paper) is depicted in Figure 7.26 in [23]. The variation of *p*_*u*_ with 1 − *h* at *α* = *π*/12 and *α* = *π*/6 has been calculated by means of (33), (34), (42), and (44). As a result, it has been found that the present solution predicts practically the same value of the extrusion pressure as the solution [23].

The general method can be extended to the process of extrusion through curvilinear dies. The complete development of the method for curvilinear dies is beyond the scope of the present paper. Therefore, its general idea is outlined below. No solution similar to that used to arrive at (42)–(44) is available for curvilinear dies. Therefore, it is of importance to evaluate the accuracy of the general method when a simple kinematically admissible velocity field is adopted instead of (35) to evaluate the extrusion pressure in the case of wedge-shaped dies. To this end, it is assumed that

(46) $$\begin{array}{c} f\left(\theta \right)=a+2b\sqrt{\alpha^{2}-\theta^{2}}, \end{array}$$

where *a* and *b* are arbitrary constants. It is evident that (46) satisfies (3). It follows from (46) that

(47) $$\begin{array}{c} F\left(\theta \right)=a\theta +b\left[\theta \sqrt{\alpha^{2}-\theta^{2}}+\alpha^{2}\arctan\left(\frac{\theta }{\sqrt{\alpha^{2}-\theta^{2}}}\right)\right], \end{array}$$

(48) $$\begin{array}{c} \frac{df}{d\theta }=-\frac{2b\theta }{\sqrt{\alpha^{2}-\theta^{2}}}. \end{array}$$

Substituting (47) at *θ* = *α* into (20) gives

(49) $$\begin{array}{c} b=\frac{2\left(\sin\alpha -a\alpha \right)}{\pi \alpha^{2}}. \end{array}$$

Substituting (46)–(49) into (34) and, then, into (33) yields the value of *p*_*u*_ which depends on *a*. Minimizing *p*_*u*_ with respect to this parameter determines the best upper bound on the value of *P* based on the kinematically admissible velocity field chosen. Numerical calculation has shown that the difference between the values of *p*_*u*_ found with the use of (42) and (46) is less than 1%. Therefore, the method allows for the use of simple kinematically admissible velocity fields for rapid analysis and design of the process of plane strain extrusion. In particular, it is evident that (46) can be modified to build up a kinematically admissible velocity field for the extrusion through a curvilinear die. For example, it is possible to guess a streamline based coordinate system such that one of the velocity components in planes of flow vanishes. Then, it is necessary to repeat all steps starting from (4) to arrive at an expression similar to (32). In particular, (4) should be found by integrating the equation of incompressibility in the curvilinear streamline based coordinate system.

## 6. Conclusions

1. A general kinematically admissible velocity field has been built up for the process of extrusion through a wedge-shaped die. This velocity field satisfies the asymptotic behavior of the real velocity field in the vicinity of maximum friction surfaces.
2. Equations for the power dissipation within the plastic zone, at the velocity discontinuities and at the friction surface, have been developed in terms of ordinary integrals. These allow the calculation of the power required for the extrusion process.
3. A semianalytic solution for flow through an infinite converging channel has been adopted to calculate an upper bound on the extrusion force by the method developed. The very close agreement between this upper bound solution and a slip-line solution found by the method of Riemann has been found.
4. A very simple kinematically admissible velocity field has been proposed to calculate an upper bound on the extrusion force by the method developed. The very close agreement between the two upper bound solutions has been found. This suggests that simple kinematically admissible velocity fields satisfying (3) can be used to find accurate upper bound solutions. This conclusion is important for the process of extrusion through curvilinear dies. No semianalytic solution is available in this case but a general kinematically admissible velocity field similar to that proposed in the present paper can be built up for such processes.
5. The method can be extended to axisymmetric extrusion. To this end, it is just necessary to account for (3) in the general kinematically admissible velocity field proposed in [12].
